# Body Mass Index and Its Influence on Chronic Low Back Pain in the Spanish Population: A Secondary Analysis from the European Health Survey (2020)

**DOI:** 10.3390/biomedicines11082175

**Published:** 2023-08-02

**Authors:** María Orosia Lucha-López, César Hidalgo-García, Sofía Monti-Ballano, Sergio Márquez-Gonzalvo, Loreto Ferrández-Laliena, Julián Müller-Thyssen-Uriarte, Ana Carmen Lucha-López

**Affiliations:** 1 Unidad de Investigación en Fisioterapia, Spin off Centro Clínico OMT-E Fisioterapia SLP, Universidad de Zaragoza, Domingo Miral s/n, 50009 Zaragoza, Spain; smonti@unizar.es (S.M.-B.); 724250@unizar.es (S.M.-G.); lferrandez@unizar.es (L.F.-L.); 732751@unizar.es (J.M.-T.-U.); 2 Unidad de Investigación en Fisioterapia, Universidad de Zaragoza, Domingo Miral s/n, 50009 Zaragoza, Spain; analucha@unizar.es

**Keywords:** body mass index, chronic low back pain, prevention, physical activity

## Abstract

Risk factors such as obesity and a sedentary lifestyle contribute to the development of chronic low back pain. Purpose: To describe how body mass index (BMI) influences the prevalence of chronic low back pain in the general Spanish population and assess this influence given other factors as sex, age, physical occupational demands, and recreational physical activity. Methods: An analytical cross-sectional design was performed based on secondary data from the European Health Survey in Spain (2020). Data on age, gender, physical occupational demands, physical activity, BMI, and presence of chronic low back pain were extracted. Results: A total of 19,716 (52.2% women) subjects with a median age of 53 years old were analyzed. Of these, 18.3% had chronic low back pain, 39% were overweight, and 16.1% were obese. The adjusted generalized linear model showed that being obese increased the odds of chronic low back pain by 1.719 times (*p* < 0.001). Being a woman increased the odds by 1.683 times (*p* < 0.001). Performing occupational tasks requiring high physical demands increased the odds by 1.403 times (*p* < 0.001). Performing physical activity in leisure time several times a week decreased the odds by 0.598 times (*p* < 0.001). For every additional year of age, the odds of chronic low back pain increased by 3.3% (*p* < 0.001). Conclusions: Obesity is related with the presence of chronic low back pain in the general Spanish population. This association persists, being the more relevant factor, after adjusting the association of BMI and chronic low back pain with other factors such as sex, age, physical occupational demands, and recreational physical activity.

## 1. Introduction

Low back pain, defined as pain in the area between the inferior margin of the 12th rib and the inferior gluteal folds [1], is one of the major causes of years spent with disability and health care costs worldwide [2]. In fact, low back pain and migraines have been identified as the leading causes of years lived with disability in high-income and middle-income nations [3].

Low back pain that persists for more than 3 months is considered chronic [4]. Chronic low back pain has a prevalence between 7 to 10% in general population (21 years or older) [5], though some authors state that it might achieve up to 23% (between 25–74 years old) [6]. Chronic low back pain prevalence has been shown to be 6.3–11.1% in the United Kingdom, 13.4% in Wales [7], 5.91% in Italy [8], 7.7% in France [9], and 13.1% in the United States of America [10]. The direct and indirect economic costs of chronic low back pain are estimated at around 2.2–2.8% of the gross domestic product of industrialized countries. In Spain, based on these estimates, costs of around EUR 13,000 million to 16,000 million per year have been calculated [11]. It has been stated that, in Spain, up to 40% of working absenteeism is caused by low back pain [11].

Demographic parameters, such as sex, age, body mass index (BMI), and type of occupational task, and lifestyle parameters, such as physical activity, have been identified among the most critical risk factors in the chronicity of acute and subacute low back pain [12]. 

The prevalence of chronic low back pain is related to age, increasing from the age of 30 years [13], and it is higher among women [14]. Because of the reality that the world population of older persons, aged 60 years or over, is expected to reach nearly 2.1 billion in 2050, doubling its size from 2017 [15], the fact that the prevalence of chronic low back pain increases with age [13] has particular importance. Hormonal mechanisms [16], increased sensitivity to pain [17] and less competent pain inhibitory mechanism [18] have been highlighted as the biological factors behind the higher prevalence of low back pain in woman. 

The prevalence of chronic low back pain has been found to be higher in individuals who have physically demanding occupational task that frequently require bending, lifting, and lumbar flexion postures [19]. Occupational tasks that require driving [20] or sitting [19] for long periods have been also related to higher rates of chronic low back pain. 

Physical inactivity is recognized as the fourth most important behavioral risk factor for mortality worldwide [21]. Physical activity, on the other hand, has proven to be one of the most successful ways to improve the general population’s health across all age groups [22]. However, exercise completion and adherence can be negatively affected in conditions that cause chronic pain. Thus, a vicious cycle of pain, inertia, sedentary behavior, and increased disability has been described [23]. Even in this context, it has been stated that most individuals with chronic pain can benefit from exercise [23]. Thus, exercise is the main piece of contemporary advice for the treatment of chronic low back pain [19]. 

A higher BMI has been established as a risk factor for developing chronic low back pain [24,25]. A BMI index greater than 30 kg/m^2^ is considered obesity. In 2016, 13% of individuals over the age of 18 in the adult world population were obese (11% of men and 15% of women). Between 1975 and 2016, the prevalence of obesity roughly tripled globally [26]. Obesity and overweight are linked to both total mortality [27] and specific causes of death such as cancer, cardiovascular disease, and respiratory illnesses. Numerous chronic diseases, including cardiovascular conditions, type 2 diabetes, and several malignancies, are more prevalent in subjects who are overweight or obese [28]. Moreover, obesity decreases postural adaptation and may lead to a rise in pain, particularly in the vertebral column [29]. This increased prevalence in obesity is related to a number of factors, such as a rise in sedentary behavior in work [30] and leisure time [31], as well as a rise in the intake of high fat and carbohydrate foods [32]. 

In this particular scenario, little attention has been paid to the weighted influence of the interaction among factors in the prevalence of chronic low back pain. Of particular interest, distinguishing the challenge posed by obesity, is the influence of BMI, considering other relevant risk factors. There is a lack of relevant recent data in the field, particularly in the Spanish population. Studies should assist in creating effective preventive and treatment measures, as well as identifying pertinent risk factors, especially modifiable factors, because several of these factors (including BMI) can be addressed by improving health habits [4]. Due to the expected increase in prevalence brought on by a growing elderly population, this topic is especially relevant.

The main purpose of this paper was to describe how BMI influences the prevalence of chronic low back pain in the general Spanish population and consider this influence given other factors, such as sex, age, physical occupational demands, and recreational physical activity.

## 2. Materials and Methods

### 2.1. Study Design

An analytical cross-sectional design was performed based on data obtained through self-reported information from the European Health Survey in Spain (2020) regarding the Spanish general population. The data used in this study were obtained from a cross-sectional nationwide survey, and all data were anonymized and public. 

### 2.2. Setting

Personal or telephone interviews were conducted by the National Statistical Institute (INE) between July 2019 and July 2020 by competent interviewers. Before beginning the interviews, the interviewers received formation courses. In these courses, the methodological concepts and theoretical considerations of the survey were explained. The procedure for administering the questionnaire and the rules for conducting the field work were also explained. 

Participants were selected by random sampling stratified by region, census sections, family living places, and by final selection of one individual in each family living place with the random Kish selection grid [33].

### 2.3. Participants

Primary data were recorded from 22,072 participants. Inclusion criteria were: non-institutionalized Spanish residents, aged between 15 and 104. 

**Figure 1:**
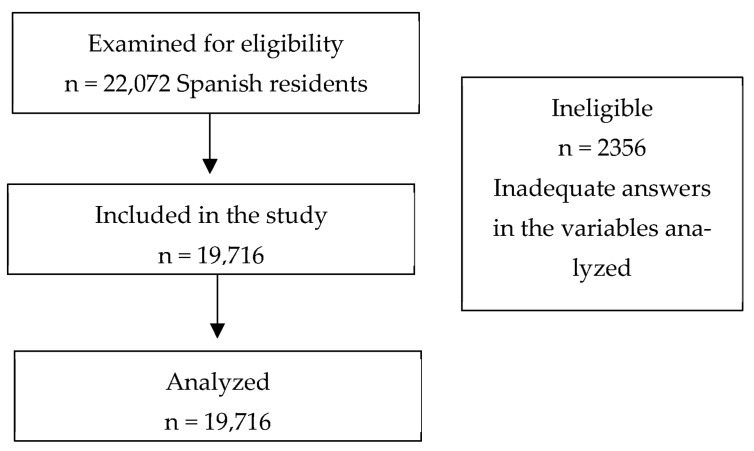
Inclusion-exclusion flow chart.

In the current analysis, individuals with missing data in some of the variables analyzed were excluded. The final sample was 19,716 individuals (Figure 1). 

### 2.4. Data Sources

We included the following variables extracted from the primary data from the European Health Survey in Spain.

#### 2.4.1. Description of the Sample

- Sex: Men/Women.
- Age: Registered in years.
- Chronic low back pain: Participants were asked if they suffered from chronic low back pain: Yes/No.
- BMI: Self-reported weight and height data were obtained in order to calculate BMI. BMI is defined as the weight in kilograms divided by the square of the height in meters. BMI was categorized according to the World Health Organization (WHO) standards (underweight: < 18.5 kg/m^2^; normal: 18.5–24.9 kg/m^2^; overweight: 25.0–29.9 kg/m^2^; obese: ≥30 kg/m^2^) [34]. Using self-reported height and weight has been classified as a valid method, with moderate to good agreement regarding direct measured anthropometric data [35].

#### 2.4.2. Physical Activity

Physical activity performed by the participants during their occupational activities and their recreational physical activity were registered. These variables were categorized as follows:

- Type of physical activity in the workplace, educational center, etc.:
  - Sitting for most of the day.
  - Standing for most of the day without making large displacements or efforts.
  - Walking, carrying some weight, making frequent displacements.
  - Performing tasks requiring high physical demand.
- Type of physical activity in leisure time:
  - I do not exercise. I spend my free time almost exclusively sedentary (read, watch TV, go to the cinema, etc.).
  - I do some occasional physical or sporting activity (walking or cycling, gardening, light gymnastics, recreational activities requiring a slight effort, etc.).
  - I do physical activity several times a month (sports, gymnastics, running, swimming, cycling, team games, etc.).
  - I do sports or physical training several times a week.

### 2.5. Statistical Methods

Sex, chronic low back pain, BMI, physical activity during occupational activities, and recreational physical activity were described offering the absolute frequencies, and the percentages in each category. Age was described with the median, 25th percentile (Q1) and 75th percentile (Q3) because it was not normally distributed according to the Kolmogorov–Smirnov test.

The chi-squared test was selected to study the relations of chronic low back pain with sex, BMI, physical activity during occupational activities, and recreational physical activity. Fisher’s Exact Test (χ^2^) for 2 × 2 tables was used when the assumptions for the chi-squared test were not accomplished [36]. The Mann–Whitney U test was used to compare age between the participants who suffered from chronic low back pain with those who did not.

Univariable generalized linear models (GLM) were constructed to model the presence of chronic low back pain as a function of each of the dependent variables independently: Sex, BMI, physical activity during occupational activities, and recreational physical activity.

One multivariable generalized linear model was constructed to model the presence of chronic low back pain as a function of all the dependent variables together. The models established the main effects considering Binomial as the distribution and Logit as the link function. The hybrid method was used for parameter estimation. Pearson chi-square was the scale parameter.

The statistical significance was established at *p* value < 0.05. SPSS 25.0 for Mac was used for the calculations.

## 3. Results

Table 1 shows the descriptive characteristics of the sample. This study includes data from 19,716 individuals, with a median age of 53 (Q1: 40.0–Q3: 67.0) years old. In this sample, 52.2% were women and 47.8% were men. The prevalence of chronic low back pain was 18.3%. A total of 42.9% had normal weight according to BMI, 39.0% were overweight, and 16.1% were obese. The majority of the sample, 44.1%, were standing for most of the day during occupational activities. In our sample, 36.2% were almost exclusively sedentary and 38.2% performed occasional physical or sporting activity during their leisure time.

**Figure 2:**
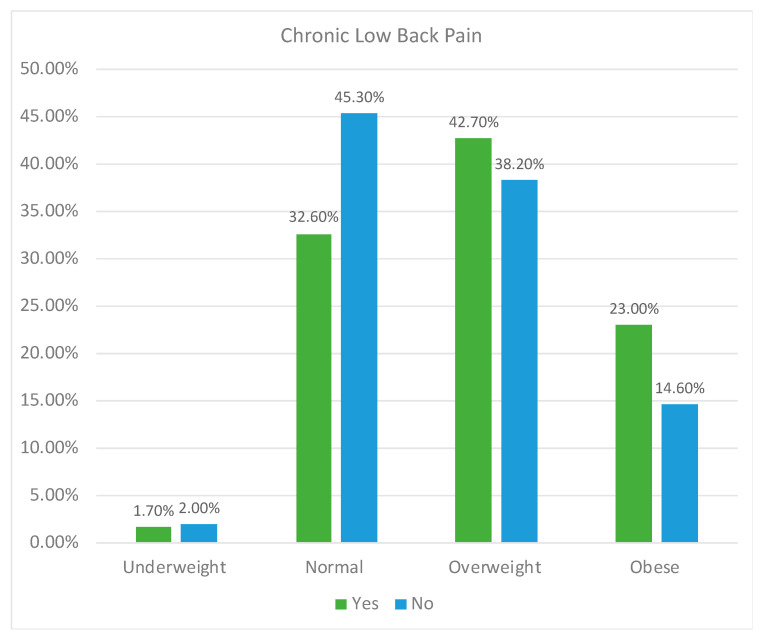
Prevalence of chronic low back pain according to BMI.

Comparative analysis of the sample according to the prevalence of low back pain is presented in Supplementary Materials Table S1. Women suffered more chronic low back pain than men (*p* < 0.001). The median age in the individuals with chronic low back pain (63.0 (Q1: 52.0–Q3: 75.0) years old) was higher than in the individuals without chronic low back pain (50.0 (Q1: 38.0–Q3: 65.0) years old) (*p* < 0.001). Participants with a BMI indicating overweight or obesity had more chronic low back pain than individuals with normal BMI or underweight individuals (*p* < 0.001) (Figure 2). Participants sitting for most of the day suffered more chronic low back pain than subjects standing, walking, or performing tasks requiring high physical demand during occupational activities (*p* < 0.001). Individuals who were almost sedentary or performing occasional physical activity had more chronic low back pain than subjects performing physical activity several times a month or several times a week (*p* < 0.001).

The outcomes of the univariable generalized linear models are presented in Table 2. The omnibus tests were significative (*p* < 0.001). The exponential value of B (Exp(B)), that is, the predicted change in the odds of suffering from chronic low back pain for a unit increase in the predictor, is also presented. Being a woman increased the odds of chronic low back pain by 1.706 times. Being obese increased the odds by 2.186 times in relation to being normal weight, and being overweight increased the odds by 1.553 times. Performing occupational tasks requiring high physical demand decreased the odds by 0.796 times, compared to sitting for most of the day. Standing without making large displacements or efforts for most of the day decreased the odds of chronic low back pain by 0.770 times compared to sitting for most of the day. Walking, carrying some weight, or frequent displacements decreased the odds of chronic low back pain by 0.651 times compared to sitting for most of the day. Performing occasional physical or sporting activity decreased the odds of chronic low back pain by 0.756 times, performing physical activity several times a month decreased the odds by 0.452 times, and performing physical activity several times a week decreased the odds by 0.363 times compared to being almost exclusively sedentary. For every additional year of age, the odds of suffering from chronic low back pain increased by 3.7%.

The goodness of fit of the multivariable generalized linear model was adequate. The dispersion coefficient showed a value close to 1 (1.017) showing no under or over dispersion of the data and the omnibus test was significative (*p* < 0.001). The generalized linear model parameter estimates for the significant categories are given in Table 3. The exponential value of B (Exp(B)), that is, the predicted change in the odds of suffering from chronic low back pain for a unit increase in each predictor, is also presented. Being a woman increased the odds of chronic low back pain by 1.683 times. Being obese increased the odds by 1.719 times in relation to being normal weight, being the most determinant factor, and being overweight increased the odds by 1.328 times. Performing occupational task requiring high physical demand increased the odds by 1.403 times compared to sitting for most of the day, and standing without making large displacements or efforts for most of the day decreased the odds of chronic low back pain by 0.869 times compared to sitting for most of the day. Performing occasional physical or sporting activity decreased the odds of chronic low back pain by 0.899 times, performing physical activity several times a month decreased the odds by 0.721 times, and performing physical activity several times a week decreased the odds by 0.598 times compared to being almost exclusively sedentary. For every additional year of age, the odds of suffering from chronic low back pain increased by 3.3%.

In the multivariable analysis, similar odds ratios were found for sex, BMI, and physical activity in leisure time with respect to the univariable analysis. In the univariable analysis, engaging in any type of physical activity during occupational activities decreased the odds compared to sitting for most of the day, though in the multivariable analysis performing occupational tasks requiring high physical demand increased the odds, and the influence of walking was not significant.

**Figure 3:**
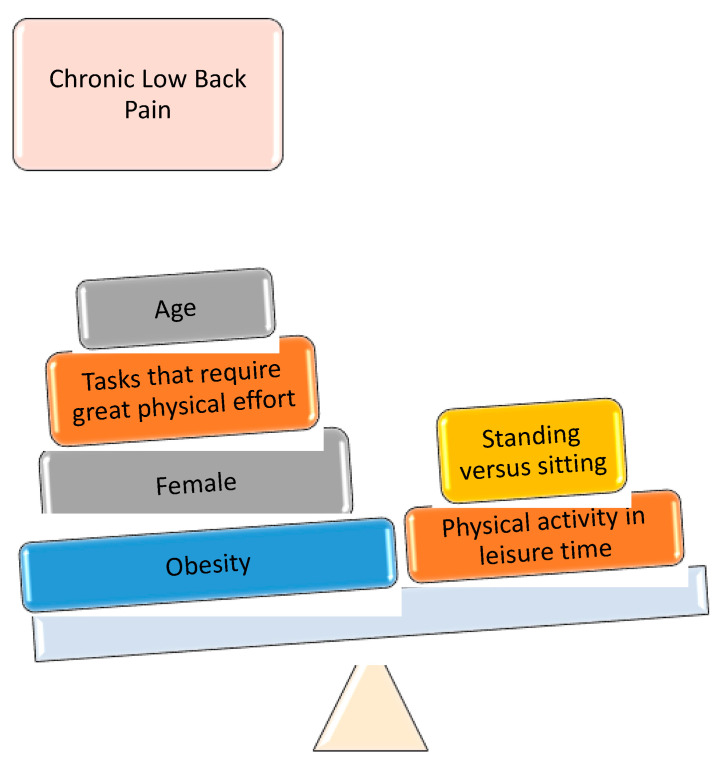
Schematic model of the multivariable analysis.

Being female and obese were found to offer the greatest odds of chronic low back pain (Figure 3).

## 4. Discussion

This study has analyzed data from 19,716 individuals, with a median age of 53 (Q1: 40.0–Q3: 67.0) years old. Though the sample was not stratified by sex, the proportion between men and women was almost balanced: 52.2% were women and 47.8% were men.

The results of this study revealed a prevalence of chronic low back pain of 18.3%. Previously, the prevalence of chronic low back pain has been established between 7% to 10% in general population [5], though some authors state that it might rise up to 23% [6]. In Spain, in 2001, the prevalence of low back pain has been stated to be 14.8% in adults of 20 years old and older (11.3% in men and 17.8% in women) [37], thus we can observe an increase in the reported prevalence. The prevalence found in this study is also higher than the prevalence reported in our geographical environment, such us the prevalence reported in France (7.7% in adults between 30–64 years old) [9], in Wales (13.4% in individuals more than 16 years old) [7], in the United Kingdom (6.3–11.1% in adults between 25–64 years old), or in Italy (5.91% in individuals more than 18 years old) [8].

A total of 42.9% had normal weight according to BMI, 39.0% were overweight, and 16.1% were obese. Previous data recorded in 2011–2012, also in the general adult population in Spain, have shown that, according to the World Health Organization recommendations, 45.0% were normal weight, 37.6% were overweight, and 17.4% were obese [38]. Our results suggest that, in terms of overweight and obesity, the adult Spanish population in 2019–2020 is similar to that in 2011–2012, though there is a slight increase in overweight and a slight decrease in obesity.

In our sample, 36.2% reported being almost exclusively sedentary and 38.2% reported performing occasional physical or sporting activity during leisure time. Data from the 2014 European Health Survey in Spain showed that 34.4% of the population aged between 18 to 74 years indicated carrying out no physical activity in leisure time and 38.9% declared they accomplished physical activity occasionally [39]. Our results are similar to those previous data, though the slight increase in sedentary behavior should be taken into consideration.

In the multivariable analysis, being a woman increased the odds of chronic low back pain by 1.683 times. As has been suggested previously, chronic low back pain is more prevalent in women, regardless of age [37,40]. It is probable that women are more likely to suffer chronic low back pain because of a number of factors, both known and unknown. Some of the relevant studied biological factors include hormonal mechanisms [16], such as variations of estrogen and/or progesterone throughout the menstrual cycle [41], the intake of hormone replacement therapy [42], and pregnancy [43], and menopause-related musculoskeletal changes, for example, osteoporosis [44]. This higher vulnerability has been ascribed, as well, to differences in genetic sensitivity [45], reflected in an increased sensitivity to pain [17] and higher susceptibility to experience temporal summation of mechanically [46] or chemically induced pain [47], and less competent pain inhibitory mechanism [18,46]. Lower efficiency in pain coping [45] has been highlighted as well.

In the multivariable analysis, being obese increased the odds of the presence of chronic low back pain by 1.719 times in relation to being normal weight, and being overweight increased the odds by 1.328 times. The associations of chronic low back pain with being obese and overweight persisted in our data even after adjusting for other variables, as in previous data from a similar population [48], though in this previous study the relation was not so strong. This must be due to the fact that the previous study analyzed data about the presence of low back pain over the previous 12 months between 2009–2012, and not about the presence of chronic low back pain, and because of the inclusion in the analysis of others factors such as marital status or educational level that we have not included. We have selected for the analysis biological determinants and health related habits that might be improved by public health prevention programs. The association between chronic low back pain and high values of BMI has also been established in a previous study in the Norwegian general population (8733 men and 10149 women, aged 30 to 69 years old) [24]. However, other researches have failed to find this association. The VISAT (VIeillissement SAnté Travail) study, in France, did not find any significant association between the occurrence of chronic low back pain and high BMI, but the studied population was composed only from workers, and they found that carrying heavy loads and other occupational factors predicted a higher risk of incidence of chronic low back pain [49]. Herin et al., also in France but with a more representative sample of workers, did not find any significant association either [50]. The theoretical background explaining the relation between low back pain and BMI includes mechanical factors, such us the overload in the spine caused by the high body weight [51] and inflammatory factors such as the relation among a pro-inflammatory environment promoted by adipose tissue and the intervertebral disc degeneration [52]. Obesity has been related to reduced disc height in the lumbar spine, particularly between the first and the fourth lumbar levels, without relation in the fifth [53]. Thus, structural changes might have a role in back pain [53]. However, heredity has also been clearly related to disc degeneration [54]. Evidence has been shown in a study on twins that genetic factors and early environment may confound the association between obesity and low back pain [55]. Moreover, the fact that low back pain may lead to obesity should be considered. Some evidence indicates that subjects with chronic low back pain tend to gain more weight than those with no pain [56], but more recent evidence suggests that cross-sectional associations between low back pain and BMI cannot be explained by inverse causality [24]. Heuch et al. stated in 2013 that the association between BMI and chronic low back pain was not explained by the effect of the pain on a later change in BMI [24]. Consequently, additional research into the nature of this relationship should be performed.

In the multivariable analysis, performing occupational tasks requiring high physical demand increased the odds of suffering from chronic low back pain by 1.403 times, compared to sitting for most of the day. Weight transporting or lifting and physical labor [57] have been related previously with chronic low back pain due to mechanical stress on neural, muscular, and joint structures in the spine [58]. However, standing without making large displacements or efforts for most of the day was a protective factor in our study compared to sitting for most of the day. This is similar to previous data which have established that the presence of low back pain increased with longer sitting times [59], but different from a recent study associating standing at work with the risk of reporting low back pain during the last 12 months compared to walking [60].

In our sample, performing physical activity in leisure time decreased the probability of chronic low back pain. The more frequent the physical activity, the smaller the probability of suffering from chronic low back pain. Though it is not clear which modality of exercise is more positive [19], a recent review and meta-analysis stated that core-based exercises might be the most beneficial for pain and function [61]. It has also been proven that a moderate degree of physical activity during non-working hours may reduce the incidence of chronic low back pain [62]. The positive outcomes in low back pain with exercise, especially core-related, might be based on the fact that fat degeneration of multifidus muscles [63] and of the common lumbosacral mass [64], as well as the smaller erector spinae cross-sectional area [65], has been found in low back pain patients. Thus, specific exercises that train these muscles might reverse the degeneration and allow better movement control, and posture control. In this way, with specific exercises, the structures of the lumbar spine would be better prepared to withstand mechanical loads. This situation is different from the mechanical loads caused by obesity or by heavy occupational tasks that may be carried out without prior training of the structures and with poor postural and movement control.

In the multivariable analysis, in our sample, the odds of suffering from chronic low back pain increased by 3.3% with every additional year of age. In the recent study by Kim et. al. in 2023, age was also a key predictor of chronic low back pain, showing increased risk from the decade of 50 to 60 years old until the decade of 80 to 90 years old [66]. Age has been related with degeneration in the structures of the spine, such us facet joins [67] or muscles [68], along with impaired balance that can contribute to the persistence of low back pain [69,70].

The above discussion might have interesting implications for the guidance of public health prevention programs on chronic low back pain, revealing the relevance of maintaining a healthy body weight to prevent chronic low back pain, as well as the necessity of performing physical activity on a regular basis, the need to include slight activity time between hours of sedentary occupational tasks, and the requirement to educate individuals in ergonomics to avoid overloading the spine during heavy occupational tasks.

### Limitations

The study presents some limitations that need to be addressed. Data has been requested from the general Spanish population; thus, the external validity might be compromised in other populations. The cross-sectional design allows associations to be established between the factors, but it is not possible to establish causal relationships. The information analyzed is based on self-reports, thus the data are sensible to possible bias. Though BMI is the international accepted measure to classify individuals in normal, overweight, or obesity categories, it does not allow differentiation between lean and fat body mass, thus it might misclassify individuals with high body weight due to muscle mass [71]. However, this study recalled data from a sample of considerable size and the factors analyzed have enabled highlighting of the relative magnitude of factors, as excessive body weight or sedentarism challenge the health of the populations in worldwide nowadays, not only for chronic low back pain, but also for a number of chronic non-transmissible diseases such as cardiovascular conditions or type 2 diabetes.

## 5. Conclusions

The results of this study lead us to conclude that obesity is related with the presence of chronic low back pain in the general Spanish population. This association persists, being the more relevant factor after adjusting the association of BMI and chronic low back pain with other factors such as sex, age, physical occupational demands, and recreational physical activity.

Due to the relevant influence of being obese in suffering from chronic low back pain, the avoidance of obesity is a priority in its prevention, especially in women, the elderly, and sedentary individuals.

## Figures and Tables

**Table 1:** Descriptive characteristics of the sample.

Characteristics (*n* = 19,716)		
**Sex**	***n* (%)**	
Men	9434 (47.8)	
Women	10,282 (52.2)	
**Chronic low back pain**	***n* (%)**	
Yes	3602 (18.3)	
No	16,114 (81.7)	
**BMI**	n (%)	
Underweight	382 (1.9)	
Normal	8467 (42.9)	
Overweight	7688 (39.0)	
Obese	3179 (16.1)	
**Physical activity during occupational activities**	***n* (%)**	
Sitting for most of the day	7596 (38.5)	
Standing for most of the day	8702 (44.1)	
Walking, carrying some weight, frequent displacements	2542 (12.9)	
Tasks requiring high physical demand	876 (4.4)	
**Physical activity in leisure time**	***n* (%)**	
Almost exclusively sedentary	7138 (36.2)	
Occasional physical or sporting activity	7538 (38.2)	
Physical activity several times a month	2005 (10.2)	
Physical activity several times a week	3035 (15.4)	
	**Median**	**Q1–Q3**
**Age (years)**	53.0	40.0–67.0

**Table 2:** Univariable generalized linear models. Parameter estimates.

Univariable Analysis. Dependent Variable: Chronic Low Back Pain	B	Crude Exp(B)	Wald 95% Confidence	Wald Chi-Square Statistic	*p* Value
			Interval for the Exp(B) Lower Bound–Upper Bound		
**Sex**					
Female	0.534	1.706	1.584–1.838	198.594	<0.001
**BMI**					
Overweight	0.440	1.553	1.429–1.688	107.692	<0.001
Obese	0.782	2.186	1.977–2.416	233.248	<0.001
**Physical activity during occupational activities**					
Standing for most of the day	−0.261	0.770	0.712–0.833	42.311	<0.001
Walking, carrying some weight, frequent displacements	−0.429	0.651	0.576–0.736	46.955	<0.001
Tasks requiring great physical effort	−0.228	0.796	0.663–0.956	5.962	0.015
**Physical activity in leisure time**					
Occasional physical or sporting activity	−0.280	0.756	0.698–0.819	47.158	0.017
Physical activity several times a month	−0.795	0.452	0.390–0.522	114.888	<0.001
Physical activity several times a week	−1.015	0.363	0.318–0.413	229.550	<0.001
**Age (years)**	0.036	1.037	1.034–1.040	644.371	<0.001

**Table 3:** Multivariable generalized linear model. Parameter estimates.

Multivariable Analysis. Dependent Variable: Chronic Low Back Pain	B	Adjusted Exp(B)	Wald 95% Confidence Interval for the Exp(B) Lower Bound–Upper Bound	Wald Chi-Square Statistic	*p* Value
Intercept	−3.644	0.026	0.022–0.031	1546.884	<0.001
**Sex**					
Female	0.521	1.683	1.552–1.825	158.861	<0.001
**BMI**					
Overweight	0.284	1.328	1.213–1.454	37.743	<0.001
Obese	0.542	1.719	1.543–1.917	95.752	<0.001
**Physical activity during occupational activities**					
Standing for most of the day	−0.141	0.869	0.797–0.948	10.040	0.002
Tasks requiring high physical demand	0.339	1.403	1.153–1.708	11.432	0.001
**Physical activity in leisure time**					
Occasional physical or sporting activity	−0.106	0.899	0.824–0.981	5.660	0.017
Physical activity several times a month	−0.327	0.721	0.617–0.842	16.942	<0.001
Physical activity several times a week	−0.515	0.598	0.519–0.688	51.160	<0.001
**Age (years)**	0.032	1,033	1.030–1.035	711.686	<0.001

## Data Availability

Data are available in the section “European Health Survey in Spain (EESE) year 2020” at https://www.ine.es, accessed on 14 December 2022.

## Supplementary Materials

The following supporting information can be downloaded at: https://www.mdpi.com/article/10.3390/biomedicines11082175/s1, Table S1: Comparative analysis of the characteristics of the sample according to the prevalence of chronic low back pain.

## References

[1] Hoy D., Brooks P., Blyth F., Buchbinder R. (2010). The Epidemiology of Low Back Pain. Best Pract. Res. Clin. Rheumatol..

[2] GBD 2017 Disease and Injury Incidence and Prevalence Collaborators (2018). Global, regional, and national incidence, prevalence, and years lived with disability for 354 diseases and injuries for 195 countries and territories, 1990–2017: A systematic analysis for the Global Burden of Disease Study 2017. Lancet.

[3] Vos T., Abajobir A.A., Abate K.H., Abbafati C., Abbas K.M., Abd-Allah F., Abdulkader R.S., Abdulle A.M., Abebo T.A., Abera S.F. (2017). Global, Regional, and National Incidence, Prevalence, and Years Lived with Disability for 328 Diseases and Injuries for 195 Countries, 1990–2016: A Systematic Analysis for the Global Burden of Disease Study 2016. Lancet.

[4] Parreira P., Maher C.G., Steffens D., Hancock M.J., Ferreira M.L. (2018). Risk Factors for Low Back Pain and Sciatica: An Umbrella Review. Spine J..

[5] Freburger J.K., Holmes G.M., Agans R.P., Jackman A.M., Darter J.D., Wallace A.S., Castel L.D., Kalsbeek W.D., Carey T.S. (2009). The Rising Prevalence of Chronic Low Back Pain. Arch. Intern. Med..

[6] Balagué F., Mannion A.F., Pellisé F., Cedraschi C. (2012). Non-Specific Low Back Pain. Lancet.

[7] Jonsdottir S., Ahmed H., Tómasson K., Carter B. (2019). Factors Associated with Chronic and Acute Back Pain in Wales, a Cross-Sectional Study. BMC Musculoskelet. Disord..

[8] Juniper M., Le T.K., Mladsi D. (2009). The Epidemiology, Economic Burden, and Pharmacological Treatment of Chronic Low Back Pain in France, Germany, Italy, Spain and the UK: A Literature-Based Review. Expert. Opin. Pharmacother..

[9] Leclerc A., Chastang J.-F., Ozguler A., Ravaud J.-F. (2006). Chronic Back Problems Among Persons 30 to 64 Years Old in France. Spine.

[10] Shmagel A., Foley R., Ibrahim H. (2016). Epidemiology of Chronic Low Back Pain in US Adults: Data From the 2009–2010 National Health and Nutrition Examination Survey. Arthr. Care Res..

[11] Castellano-Tejedor C., Costa-Requena G., Lusilla-Palacios P., Barnola-Serra E. (2014). Calidad de Vida En Pacientes Con Dolor Lumbar Crónico. Apunt. Psicol..

[12] Sadeghi-Yarandi M., Ghasemi M., Ghanjal A., Sepandi M., Soltanzadeh A. (2022). The Prediction of Chronicity in Patients with Acute and Subacute Nonspecific Low Back Pain and Associated Risk Factors: A Case-Control Study. Pain Manag. Nurs..

[13] Wong C.K.W., Mak R.Y.W., Kwok T.S.Y., Tsang J.S.H., Leung M.Y.C., Funabashi M., Macedo L.G., Dennett L., Wong A.Y.L. (2022). Prevalence, Incidence, and Factors Associated with Non-Specific Chronic Low Back Pain in Community-Dwelling Older Adults Aged 60 Years and Older: A Systematic Review and Meta-Analysis. J. Pain.

[14] Thomas E., Silman A.J., Croft P.R., Papageorgiou A.C., Jayson M.I.V., Macfarlane G.J. (1999). Predicting Who Develops Chronic Low Back Pain in Primary Care: A Prospective Study. BMJ.

[15] Man G.-M., Man M. (2018). Demographic Changes in Today’s Society Reflected in the Organizational Context. Land Forces Acad. Rev..

[16] Bailey A. (2009). Risk Factors for Low Back Pain in Women: Still More Questions to Be Answered. Menopause.

[17] Rollman G.B., Lautenbacher S. (2001). Sex Differences in Musculoskeletal Pain. Clin. J. Pain.

[18] Ge H.-Y., Madeleine P., Arendt-Nielsen L. (2004). Sex Differences in Temporal Characteristics of Descending Inhibitory Control: An Evaluation Using Repeated Bilateral Experimental Induction of Muscle Pain. Pain.

[19] Essman M., Lin C.Y. (2022). The Role of Exercise in Treating Low Back Pain. Curr. Sports Med. Rep..

[20] Tinitali S., Bowles K.-A., Keating J.L., Haines T. (2019). Sitting Posture During Occupational Driving Causes Low Back Pain; Evidence-Based Position or Dogma? A Systematic Review. Hum. Factors.

[21] Kohl H.W., Craig C.L., Lambert E.V., Inoue S., Alkandari J.R., Leetongin G., Kahlmeier S. (2012). The Pandemic of Physical Inactivity: Global Action for Public Health. Lancet.

[22] Bull F.C., Al-Ansari S.S., Biddle S., Borodulin K., Buman M.P., Cardon G., Carty C., Chaput J.-P., Chastin S., Chou R. (2020). World Health Organization 2020 Guidelines on Physical Activity and Sedentary Behaviour. Br. J. Sports Med..

[23] Borisovskaya A., Chmelik E., Karnik A., Xiao J. (2020). Exercise and Chronic Pain. Physical Exercise for Human Health.

[24] Heuch I., Heuch I., Hagen K., Zwart J.-A. (2013). Body Mass Index as a Risk Factor for Developing Chronic Low Back Pain: A Follow-up in the Nord-Trøndelag Health Study. Spine.

[25] Leboeuf-Yde C. (2000). Body Weight and Low Back Pain: A Systematic Literature Review of 56 Journal Articles Reporting on 65 Epidemiologic Studies. Spine.

[26] World Health Organization Obesity and Overweight. https://www.who.int/health-topics/obesity#tab=tab_1.

[27] Bhaskaran K., Dos-Santos-Silva I., Leon D.A., Douglas I.J., Smeeth L. (2018). Association of BMI with Overall and Cause-Specific Mortality: A Population-Based Cohort Study of 3·6 million Adults in the UK. Lancet Diabetes Endocrinol..

[28] Guh D.P., Zhang W., Bansback N., Amarsi Z., Birmingham C.L., Anis A.H. (2009). The Incidence of Co-Morbidities Related to Obesity and Overweight: A Systematic Review and Meta-Analysis. BMC Public Health.

[29] Gilleard W., Smith T. (2007). Effect of Obesity on Posture and Hip Joint Moments during a Standing Task, and Trunk Forward Flexion Motion. Int. J. Obes..

[30] Baradaran Mahdavi S., Riahi R., Vahdatpour B., Kelishadi R. (2021). Association between Sedentary Behavior and Low Back Pain; A Systematic Review and Meta-Analysis. Health Promot. Perspect..

[31] Tricás-Vidal H.J., Lucha-López M.O., Hidalgo-García C., Vidal-Peracho M.C., Monti-Ballano S., Tricás-Moreno J.M. (2022). Health Habits and Wearable Activity Tracker Devices: Analytical Cross-Sectional Study. Sensors.

[32] Popkin B.M., Hawkes C. (2016). Sweetening of the Global Diet, Particularly Beverages: Patterns, Trends, and Policy Responses. Lancet Diabetes Endocrinol..

[33] Kish L. (1949). A Procedure for Objective Respondent Selection within the Household. J. Am. Stat. Assoc..

[34] Bassuk S.S., Manson J.A.E. (2005). Epidemiological Evidence for the Role of Physical Activity in Reducing Risk of Type 2 Diabetes and Cardiovascular Disease. J. Appl. Physiol..

[35] Huang D., Huang Y., Khanna S., Dwivedi P., Slopen N., Green K.M., He X., Puett R., Nguyen Q. (2020). Twitter-Derived Social Neighborhood Characteristics and Individual-Level Cardiometabolic Outcomes: Cross-Sectional Study in a Nationally Representative Sample. JMIR Public Health Surveill..

[36] Papadimitriou K., Loupos D. (2021). The Effect of an Alternative Swimming Learning Program on Skills, Technique, Performance, and Salivary Cortisol Concentration at Primary School Ages Novice Swimmers. Healthcare.

[37] Carmona L., Ballina J., Gabriel R., Laffon A. (2001). The Burden of Musculoskeletal Diseases in the General Population of Spain: Results from a National Survey. Ann. Rheum. Dis..

[38] Arrospide A., Machón M., Ramos-Goñi J.M., Ibarrondo O., Mar J. (2019). Inequalities in Health-Related Quality of Life According to Age, Gender, Educational Level, Social Class, Body Mass Index and Chronic Diseases Using the Spanish Value Set for Euroquol 5D-5L Questionnaire. Health Qual. Life Outcomes.

[39] Fernandez-Navarro P., Aragones M.T., Ley V. (2018). Leisure-Time Physical Activity and Prevalence of Non-Communicable Pathologies and Prescription Medication in Spain. PLoS ONE.

[40] Kopec J.A., Sayre E.C., Esdaile J.M. (2004). Predictors of Back Pain in a General Population Cohort. Spine.

[41] Cairns B.E., Gazerani P. (2009). Sex-Related Differences in Pain. Maturitas.

[42] Musgrave D.S., Vogt M.T., Nevitt M.C., Cauley J.A. (2001). Back Problems among Postmenopausal Women Taking Estrogen Replacement Therapy: The Study of Osteoporotic Fractures. Spine.

[43] To W.W.K., Wong M.W.N. (2003). Factors Associated with Back Pain Symptoms in Pregnancy and the Persistence of Pain 2 Years after Pregnancy. Acta Obstet. Gynecol. Scand..

[44] Wong A.Y.L. (2016). Musculoskeletal Pain in Postmenopausal Women—Implications for Future Research. Hong Kong Physiother. J..

[45] Staud R., Robinson M.E., Vierck C.J., Price D.D. (2003). Diffuse Noxious Inhibitory Controls (DNIC) Attenuate Temporal Summation of Second Pain in Normal Males but Not in Normal Females or Fibromyalgia Patients. Pain.

[46] Sarlani E., Greenspan J.D. (2002). Gender Differences in Temporal Summation of Mechanically Evoked Pain. Pain.

[47] Ge H.Y., Madeleine P., Arendt-Nielsen L. (2005). Gender differences in pain modulation evoked by repeated injections of glutamate into the human trapezius muscle. Pain.

[48] Palacios-Ceña D., Alonso-Blanco C., Hernández-Barrera V., Carrasco-Garrido P., Jiménez-García R., Fernández-de-las-Peñas C. (2015). Prevalence of Neck and Low Back Pain in Community-Dwelling Adults in Spain: An Updated Population-Based National Study (2009/10–2011/12). Eur. Spine J..

[49] Esquirol Y., Niezborala M., Visentin M., Leguevel A., Gonzalez I., Marquié J.-C. (2017). Contribution of Occupational Factors to the Incidence and Persistence of Chronic Low Back Pain among Workers: Results from the Longitudinal VISAT Study. Occup. Environ. Med..

[50] Herin F., Vézina M., Thaon I., Soulat J.-M., Paris C., ESTEV Group (2014). Predictive Risk Factors for Chronic Regional and Multisite Musculoskeletal Pain: A 5-Year Prospective Study in a Working Population. Pain.

[51] Samartzis D., Karppinen J., Chan D., Luk K.D.K., Cheung K.M.C. (2012). The Association of Lumbar Intervertebral Disc Degeneration on Magnetic Resonance Imaging with Body Mass Index in Overweight and Obese Adults: A Population-Based Study. Arthritis Rheum..

[52] Ruiz-Fernández C., Francisco V., Pino J., Mera A., González-Gay M.A., Gómez R., Lago F., Gualillo O. (2019). Molecular Relationships among Obesity, Inflammation and Intervertebral Disc Degeneration: Are Adipokines the Common Link?. Int. J. Mol. Sci..

[53] Urquhart D.M., Kurniadi I., Triangto K., Wang Y., Wluka A.E., O’Sullivan R., Jones G., Cicuttini F.M. (2014). Obesity Is Associated with Reduced Disc Height in the Lumbar Spine but Not at the Lumbosacral Junction. Spine.

[54] Videman T., Battié M.C., Ripatti S., Gill K., Manninen H., Kaprio J. (2006). Determinants of the Progression in Lumbar Degeneration: A 5-Year Follow-up Study of Adult Male Monozygotic Twins. Spine.

[55] Dario A.B., Ferreira M.L., Refshauge K.M., Lima T.S., Ordoñana J.R., Ferreira P.H. (2015). The Relationship between Obesity, Low Back Pain, and Lumbar Disc Degeneration When Genetics and the Environment Are Considered: A Systematic Review of Twin Studies. Spine J..

[56] Lake J.K., Power C., Cole T.J. (2000). Back Pain and Obesity in the 1958 British Birth Cohort: Cause or Effect?. J. Clin. Epidemiol..

[57] Campbell C., Muncer S.J. (2005). The Causes of Low Back Pain: A Network Analysis. Soc. Sci. Med..

[58] Eriksen W., Natvig B., Bruusgaard D. (1999). Smoking, Heavy Physical Work and Low Back Pain: A Four-Year Prospective Study. Occup. Med..

[59] Park S.-M., Kim H.-J., Jeong H., Kim H., Chang B.-S., Lee C.-K., Yeom J.S. (2018). Longer Sitting Time and Low Physical Activity Are Closely Associated with Chronic Low Back Pain in Population over 50 Years of Age: A Cross-Sectional Study Using the Sixth Korea National Health and Nutrition Examination Survey. Spine J..

[60] Leivas E.G., Corrêa L.A., Nogueira L.A.C. (2022). The Relationship between Low Back Pain and the Basic Lumbar Posture at Work: A Retrospective Cross-Sectional Study. Int. Arch. Occup. Environ. Health.

[61] Owen P.J., Miller C.T., Mundell N.L., Verswijveren S.J.J.M., Tagliaferri S.D., Brisby H., Bowe S.J., Belavy D.L. (2020). Which Specific Modes of Exercise Training Are Most Effective for Treating Low Back Pain? Network Meta-Analysis. Br. J. Sports Med..

[62] Solovev A., Watanabe Y., Kitamura K., Takahashi A., Kobayashi R., Saito T., Takachi R., Kabasawa K., Oshiki R., Platonova K. (2020). Total Physical Activity and Risk of Chronic Low Back and Knee Pain in Middle-Aged and Elderly Japanese People: The Murakami Cohort Study. Eur. J. Pain.

[63] Chan S.-T., Fung P.-K., Ng N.-Y., Ngan T.-L., Chong M.-Y., Tang C.-N., He J.-F., Zheng Y.-P. (2012). Dynamic Changes of Elasticity, Cross-Sectional Area, and Fat Infiltration of Multifidus at Different Postures in Men with Chronic Low Back Pain. Spine J..

[64] Goubert D., De Pauw R., Meeus M., Willems T., Cagnie B., Schouppe S., Van Oosterwijck J., Dhondt E., Danneels L. (2017). Lumbar Muscle Structure and Function in Chronic versus Recurrent Low Back Pain: A Cross-Sectional Study. Spine J..

[65] Sions J.M., Elliott J.M., Pohlig R.T., Hicks G.E. (2017). Trunk Muscle Characteristics of the Multifidi, Erector Spinae, Psoas, and Quadratus Lumborum in Older Adults with and Without Chronic Low Back Pain. J. Orthop. Sports Phys. Ther..

[66] Kim J.G., Park S.-M., Kim H.-J., Yeom J.S. (2023). Development and Validation of a Risk-Prediction Nomogram for Chronic Low Back Pain Using a National Health Examination Survey: A Cross-Sectional Study. Healthcare.

[67] Bo Y., Kaibiao J., Xinfeng L., Jidong Z., Zude L. (2017). Correlation of the Features of the Lumbar Multifidus Muscle with Facet Joint Osteoarthritis. Orthopedics.

[68] Lee D.G., Bae J.H. (2023). Fatty Infiltration of the Multifidus Muscle Independently Increases Osteoporotic Vertebral Compression Fracture Risk. BMC Musculoskelet. Disord..

[69] Xiao F., Maas H., van Dieën J.H., Pranata A., Adams R., Han J. (2022). Chronic Non-Specific Low Back Pain and Ankle Proprioceptive Acuity in Community-Dwelling Older Adults. Neurosci. Lett..

[70] Ito T., Sakai Y., Yamazaki K., Igarashi K., Sato N., Yokoyama K., Morita Y. (2017). Proprioceptive Change Impairs Balance Control in Older Patients with Low Back Pain. J. Phys. Ther. Sci..

[71] Andreoli A., Garaci F., Cafarelli F.P., Guglielmi G. (2016). Body Composition in Clinical Practice. Eur. J. Radiol..

