# Supplementary material for: Body Mass Index and Its Influence on Chronic Low Back Pain in the Spanish Population: A Secondary Analysis from the European Health Survey (2020)
Source: Biomedicines. 2023 Aug 2;11(8):2175. doi: 10.3390/biomedicines11082175 (PMC10452522; doi:10.3390/biomedicines11082175)
Supplement: Supplementary file 1 [file biomedicines-11-02175-s001.zip › biomedicines-2505061-supplementary.pdf]

Table S1. Comparative analysis of the characteristics of the sample according to the prevalence of chronic low back pain

**Characteristics (n=19,716)**	**Chronic Low Back Pain**		
	**Yes**	**No**	***p* value**
**Sex**			
Men	37.2%	50.2%	<0.001
Women	62.8%	49.8%	
**BMI**			
Underweight	1.7%	2.0%	<0.001
Normal	32.6%	45.3%	
Overweight	42.7%	38.2%	
Obese	23.0%	14.6%	
**Physical activity during occupational activities**			
Sitting for most of the day	44.3%	37.2%	<0.001
Standing for most of the day	41.1%	44.8%	
Walking, carrying some weight, frequent displacements	10.4%	13.4%	
Tasks requiring high physical demand	4.2%	4.5%	
**Physical activity in leisure time**			
Almost exclusively sedentary	46.0%	34.0%	<0.001
Occasional physical or sporting activity	39.0%	38.1%	
Physical activity several times a month	6.7%	10.9%	
Physical activity several times a week	8.3%	17.0%	
	**Median (Q1-Q3)**	**Median (Q1-Q3)**	
**Age (years)**	63.0 (52.0- 75.0)	50.0 (38.0- 65.0)	<0.001
